# Supplementary material for: ChatGPT as a Tool for Biostatisticians: A Tutorial on Applications, Opportunities, and Limitations
Source: Stat Med. 2025 Oct 23;44(23-24):e70263. doi: 10.1002/sim.70263 (PMC12548020; doi:10.1002/sim.70263)
Supplement: Supplementary file 1 — Data S1. Supporting Information. [file SIM-44-0-s001.zip › Supplementary Materials Additional.zip!/3.1_Systematic_Reviews_and_Meta_aspects/3.1_Data Extraction - o4-mini - Chat 7.pdf]

Use Case Meta-Analysis - Data Extraction - o4-mini - Chat 7

Rudloff et al. - 2009 - Concurrent lobular neoplasia increases the risk of ipsilateral breast cancer recurrence in situ treated with breast-conserving therapy.pdf
PDF

Vidali et al. - 2012 - Conservative treatment of breast ductal carcinoma in situ results of an Italian multi-institutional.pdf
PDF

The aim is to perform a meta-analysis on total ipsilateral local recurrence rates at 10 years in cases of ductal carcinoma in situ.

Data inclusion rules are: To ensure the validity of the follow-up data, the results should be as close to 10 years follow-up (minimum) as the information in each study allows. If there is more than one publication from an institution or group, the most recent study with appropriate follow-up will be used to extract 10-year data.

Please provide from each of the two attached studies:

- the rate of ipsilateral local recurrence for conservative surgery with additional radiotherapy (no other interventions or drugs allowed) after 10 years

- the number of eligible patients

- period of data collection

- duration of follow-up

- type of study

- age of patients (mean or median, maximum and minimum)

- treatment modalities

For this task you have access to up-to-the-minute web retrieval to find adequate approaches for data extraction. When answering the task above please proceed as follows:

1. Fetch the most relevant sources.

2. Show your step-by-step reasoning.

3. At the end, give me a concise answer citing the sources you used.

4. Please provide a dataframe with one entry for each study, and column names "study", "rate", "n_patients", "period", "follow_up", "study_type", "age", and "treatment".

Step-by-Step Reasoning

1. **Identify the cohort of interest**
   Both studies report outcomes for patients with DCIS treated by breast-conserving surgery **plus** adjuvant external-beam radiotherapy, without other systemic therapies.
2. **Extract key details from Rudloff et al. 2009**
   - **Period of data collection:** 1991–1995.
   - **Eligible patients:** 91 of 294 received radiotherapy (no chemotherapy; 6.1% received tamoxifen but chemotherapy was prohibited) .
   - **Duration of follow-up:** median 11.8 years for those who received radiation .
   - **10-year ipsilateral local recurrence rate:** 11.9% for patients treated with surgery + radiation .
   - **Type of study:** Single-institution retrospective cohort.
   - **Age:** median 55 years (range 26–89) .
   - **Treatment modalities:** breast-conserving excision + external-beam tangential whole-breast irradiation (50 Gy median), no chemotherapy; tamoxifen optional in 6.1% .
3. **Extract key details from Vidali et al. 2012**
   - **Period of data collection:** February 1985–March 2000.
   - **Eligible patients:** 586 women treated (all underwent conservative surgery + whole-breast RT) .
   - **Duration of follow-up:** median 136 months (~11.3 years; range 16–292 months) .
   - **10-year ipsilateral local recurrence rate:** 9.6% .
   - **Type of study:** Multi-institutional retrospective cohort.
   - **Age:** median 55 years (range 29–84) .
   - **Treatment modalities:** breast-conserving surgery (quadrantectomy/tumorectomy/wide excision) followed by whole-breast irradiation (median 50 Gy) with boost in 50% (median 10 Gy) .

---

study	rate	n_patients	period	follow_up	study_type	age
Rudloff et al. (2009)	11.9%	91	1991–1995	median 11.8 y	Retrospective single-institution	median 55 y (26–89)
Vidali et al. (2012)	9.6%	586	Feb 1985–Mar 2000	median 136 mo (11.3 y)	Retrospective multi-institutional	median 55 y (29–84)

Citations:
Rudloff et al.—period, eligible n, follow-up, RT group recurrence rate, age, treatment

Vidali et al.—period, n, follow-up, recurrence rate, age, treatment

Quellen
